# Use of a Teledentistry-based Program for Screening of Early Childhood Caries in a School Setting

**DOI:** 10.7759/cureus.1416

**Published:** 2017-07-01

**Authors:** Subbalekshmi T, Vasanthakumari Anandan, Renugalekshmi Apathsakayan

**Affiliations:** 1 Pediatric Dentistry, Sri ramachandra University; 2 Paediatric Dentistry, Adhiparasakthi Dental College & Hospital; 3 Paediatric Dentistry, Al Jassan University

**Keywords:** early childhood caries, school health program, caries screening, teledentistry

## Abstract

Aim

The aim of this study was to assess the reliability and feasibility of using teledentistry for the screening and diagnosis of dental caries in children between the age groups of three to six years.

Design

This study included a total of 318 school-going children whose caries scores were calculated by visual method and using digital photographs generated by an intraoral camera by two examiners: examiner 1 and examiner 2 (E1 and E2). Intra-examiner and inter-examiner variability were determined. Reliability was compared across the three groups.

Results

Intra-examiner and inter-examiner variability when compared revealed no significant difference. A Cronbach’s alpha of 0.983 was generated, which shows high reliability.

Conclusions

Effective screening for early childhood caries (ECC) in young children was possible with digital images generated in a school setting, thus paving the way for the application of teledentistry as effective means for the diagnosis of dental caries.

## Introduction

Dental caries is the most prevalent and common dental affliction of childhood. Above being a common disease, when left without treatment, caries causes complications such as toothache and dentoalveolar abscess, which could become a burden to the parents and guardians [1]. In spite of dental caries being a preventable disease and credible scientific advances being made toward its prevention, it continues to pose as a major health problem. Schools can be an important setting for health education programs, controlling the growing burden of oral diseases and promoting oral health and preventive programs [2]. The school dental health programs are aimed at improving and motivating the parents as well as their children toward oral health and treatment needs [3]. The major roadblock to applying school-based oral health programs has been the physical distance between the dentist and the patients [4-5].

Teledentistry strives to combine telecommunication technology and dental care [5-6]. The term "teledentistry" was used in 1997, when Cook defined it as "the practice of using video-conferencing technologies to diagnose and provide advice about treatment over a distance [7]." Telemedicine has a variety of applications in patient care, education, research, administration, and public health along with advantages like ease of access to remote areas, time conservation, and costs of transporting the patient [8]. Monitoring home care and ambulatory monitoring of patients can be done using telemedicine [9-10]. Telemedicine improves communication between health providers who were relatively inaccessible before.

Mainly two types of telehealth programs are practised: ‘the store and forward method’ and the ‘videoconferencing method.’ The former is used in case of non-emergency situations when images and information are collected and mailed to the specialist for consultation. This is the most commonly used system in dentistry and has found an effective use for orthodontic consultations. The latter method involves the use of videoconferencing equipment at both locations for a ‘real-time’ consultation to take place.

Although telemedicine programs have been in place for more than the last 40–50 years, the use of this technology in dentistry is very minimal [1, 7]. The major applications of teledentistry programs have been for specialist referrals and for consultations [8]. There have been previous efforts to use this technology for the diagnosis of pathological conditions [9]. For a long time, dental caries detection has been done by visual and tactile examination. Few studies have evaluated the use of intraoral photographs or digital images for the diagnosis of dental caries [11]. The use of intraoral cameras in the epidemiological setting has been shown to be acceptable to children [12]. An advantage that the use of intraoral photographs has over visual examination methods in such studies is the ability to archive intraoral photographs. This permits multiple scorers to score the images as well as remote scoring and longitudinal analysis [13].

A program for promoting dental health should be designed such that it is accessible to all, less time-consuming, cost-effective, and cause minimal disruption of daily routines. School-based dental health programs serve most of these criteria, but for the fact that a dentist has to be present physically at school during school hours. The combination of teledentistry programs and school dental health programs could be the ideal answer for overcoming the major barriers to achieving a head start toward oral health care. It is in line with this idea that this study was initiated to assess the reliability and feasibility of using teledentistry for screening and diagnosis of dental caries in children between the age groups of three and six years.

## Materials and methods

The present study was conducted in three different schools in and around Porur, Chennai. The study population consisted of three- to six-year-old healthy school-going children. All children studying in lower kindergarten (LKG), upper kindergarten (UKG), and first standard (I^st^ Standard) were invited to participate in this study (total of 318). Children within six years of age whose permanent teeth had erupted and children who were uncooperative during the procedure even after behavior modifications were excluded from this study.

Ethical approval

This study commenced after a letter of information regarding objective, time, date, and procedure of this study was circulated to the three schools and permission was issued by the respective heads of the institution. Parental consent was obtained for all the children who participated in this study (312).

The study population (312 children) was divided into three groups as per year of study at school, Group I—students studying in LKG (three- to four-year-olds), Group II—students studying in UKG (four- to five-year-olds), and Group III—students studying in I^st^ standard (five- to six-year-olds).

Calibration of examiners

Intra-examiner Reproducibility

Five patients, not related to the study, whose decayed, missing, filled teeth (dmft) was recorded with visual examination and with photographs were used as calibration for the examiner. Examiner 1 and examiner 2 (E1 and E2) examined the patients in person on two separate occasions, 48 hours apart. The calibration was accepted if the results of the measurements at baseline and at 48 hours were the same in more than 90% of the cases.

*Inter-examiner* *Calibration*

Similarly, both examiners observed photographs of five patients separately and calculated dmft. The calibration was accepted if the examiners' scores were the same in more than 90% of the cases at baseline and at 48 hours.

Phase I - gold standard phase

This study was conducted in two phases. In phase I, E1 examined the children under the light of the intraoral camera and dmft was noted. E1 also made photographs of the teeth of the each child using a 2.5 megapixel intraoral camera (Dr. Schwartz Home Care Intraoral Camera, Japan). The children were examined in their respective schools. In each quadrant, the molars were photographed separately. The lingual and labial aspects of mandibular and maxillary anteriors were taken separately. A total of eight intraoral images were generated for each patient. A proforma was prepared to collect the data regarding the oral health status and general information. This included information about age, year of study, relevant medical history, and history of previous dental visits.

The index of choice was decayed, missing, filled teeth index (dmft) as all children were in their primary dentition. The index used was the dmft index put forth by Gruebbel AO in 1944, which is the most accepted and used index for primary teeth caries evaluation. A tooth was considered decayed (D) if there was visible evidence of cavitation. This included teeth with untreated dental caries and filled teeth with recurrent caries. Teeth with restorations were scored as filled (F). The missing component (M) included only those teeth lost due to caries. In case of missing anterior teeth, it was ascertained by asking the children if the tooth loss was following trauma or due to caries and scored accordingly by the examiner. All the teeth are scored and the dmft score of a child could range from 0 to 20.

Phase II

The images captured at school were transferred to a computer. Then, E1 examined the images on the computer LCD 15-inch screen with resolution of 1,440 × 900 and scored the teeth for each student. These dmft values were calculated after a washout period of two weeks. The images of the teeth were examined separately by another examiner designated as E2 on the same computer. Three values of dmft index scores were generated for every patient who was screened: dmft1—as examined and scored by E1 in gold standard phase, dmft2—as examined and scored by E1 (Image), and dmft3—as examined and scored by E2 (Image).

Statistical analysis

Data was analyzed by using Statistical Package for the Social Sciences (SPSS) 19.0 (IBM Corp. NY, USA). The mean differences in dmft scores between age groups when determined by different methods were compared using the Post Hoc Test for multiple comparisons and Tukey's Honest Significant Difference (HSD). Reliability was assessed by generating Cronbach's Alpha. Inter-examiner reliability and intra-examiner reliability were used to compare variables. The Fischer’s Exact Test was used to compare the dmft scores dmft1, dmft2, and dmft3 for each age group.

## Results

A total of 318 children were screened for this study. Six samples (2%) were excluded due to uncooperativeness to examination, and the total number of samples that fulfilled all the inclusion criteria was 312 (98%). The total study population was further divided into three groups, of which 102 children were in Group I (LKG), 106 children were in the Group II (UKG), and 102 children were in Group III (I^st^ Standard).

As a test for internal consistency, Cronbach’s Alpha was calculated. Table 1 compares the dmft scored by E1 on visual examination (dmft1), based on intraoral photographs (dmft2) and E2 (dmft3). A *p*-value of less than 0.001 was taken as being 99.9% significant.

**Table 1:** Comparison of dmft1, dmft2, and dmft3 SD: Standard Deviation, CI Confidence Interval.

	Mean	SD	Cronbach’s Alpha	*p*-value	ICC	95% CI
dmft_1_ V/S dmft_2_	3.32	3.415	.971	.000***	.943	.929 to .954
	3.32	3.185				
dmft_1_ V/S dmft_3_	3.32	3.415	.982	.000***	.965	.956 to .972
	3.44	3.193				
dmft_2_ V/S dmft_3_	3.32	3.185	.972	.000***	.946	.933 to .956
	3.44	3.193				
dmft_1_ V/S dmft_2_ V/S dmft_3_	3.32	3.415	.983	.000***	.951	.942 to .960
	3.32	3.185				
	3.44	3.193				

Table 2 shows mean differences in dmft scores between the age groups determined by different methods compared using the Post Hoc Test for multiple comparisons. When the dmft scored by E1 in visual examinations were compared, there was an insignificant mean difference between Group II and Group III, i.e., between four- to five-year-olds and five- to six-year-olds. This trend was further seen for scores dmft2 and dmft3 across age groups where the insignificant difference was seen between Group I and Group III, while differences between all the groups were significant.

**Table 2:** Comparison of the dmft scores based on different age groups SD: Standard Deviation

		*N*	Mean	SD	*T*	*p*-value
Group I 3–4 Years	dmft1	103	2.16	2.704	20.166	.000***
	dmft2		2.15	2.483		
	dmft3		2.33	2.447		
Group II 4–5 Years	dmft1	105	3.94	3.724	22.084	.000***
	dmft2		3.92	3.583		
	dmft3		4.03	3.601		
Group III 5–6 Years	dmft1	101	3.86	3.453	58.54	.000***
	dmft2		3.89	3.079		
	dmft3		3.97	3.145		

## Discussion

Telemedicine is the use of telecommunication and information technologies in order to provide clinical health care at a distance [9-11]. In spite of the fact that caries is preventable and that efforts have been on for the past few decades to stop the advance of this disease, there is still much to be desired [14]. Also, issues like the large rural base of developing countries and very low dentist-to-patient ratio need to be considered when strategies for caries prevention are designed.

The methodology used in this study has been put to test in a few studies till date [14-16]. The study population consisted of three- to six-year-old healthy school-going children. The children were all from a semi-urban population. All the children included in this study were of South Indian origin. Of the three schools, which were made part of this study, one was a government school where the students were predominantly from a lower socioeconomic group. The other two schools were aided schools where the children belonged to middle-income group families. This age group was considered because it has been seen in the previous work by Meera, et al. that the average age of the first dental visit was only above the age of six years in Chennai; hence, we aimed at making dental care accessible at an earlier age [17].

In studies by Kopycka-Kedzierawski, et al., images were taken and transferred via the internet for immediate diagnosis [15-16]. The store and forward technology has been used here, where photographs of patients' teeth were taken and then analyzed later after being transferred to a computer. The quality and resolution of the used camera could be considered as superior as the number of samples that were excluded due to errors and non-clarity of images was negligible. A dentist made the intraoral pictures in this study, unlike in studies where allied health science graduates or primary school caretakers were trained to make pictures using an intraoral camera [14-16]. The pictures were made by a dentist as utilization of any other personnel required special training. Also, since the dentist had to examine the children for evaluating the dmft scores, the same person taking the pictures reduced the time spent with each child during the examination.

In previous studies, only intra-examiner variance has been calculated for assessing the feasibility of teledentistry [16]. In this study, both intra- and inter-examiner variance were calculated. The lowest reliability estimate was seen in Group I, this could be because of the disagreement between examiners due to some difference in the quality of images or because of the fact that this group had the least dmft scores, which implies that even minor variations in scoring could have had a pronounced effect on the resultant value. The dmft score was seen to be higher in patients with a past dental history. The highest number of children with a past dental history was noted to be in Group II. The elevation of dmft value in this group was due to the higher number of restored teeth in these children. Also, it was noticed that children with a past dental history were more cooperative to the examination.

In a school setting, children are under peer pressure, and hence elicit a more cooperative response [18]. This was well evident by the fact that only less than 5% of the population was not very cooperative. Three children had to be excluded from this study because they were uncooperative. All the three children belonged to Group I (LKG) students. Of the three, two belonged to the same school and were very uncooperative to the procedure even after behavior modification techniques like Tell Show Do and modeling were done. The study population also included children with special health care needs like children with auditory aids and physically challenged children. All of these children were found to be cooperative to the procedure after desensitization with modeling and Tell Show Do. As E1 was a pediatric dentist, the examiner applied behavior modification techniques as and when necessary to facilitate better cooperativeness for the procedure. The major advantage of school-based dental health program is that long-term evaluation is possible, cost effective, and time saving. Also, children would not require losing school hours to visit a dentist [18].

There are some limitations inherent to this study. The major limitation with reference to the methodology is the use of the store and forward method where the loss of image clarity due to transfer was not calculated. That could play a major role in diagnosis. Also, dental caries were diagnosed only with visual examinations, which would have yielded false positives, and thus increased the dmft scores. In the diagnosis of dental caries without radiographs or tactile examination, there is a possibility of missing proximal caries where there has been no breakdown of tooth structure. This would lead to an underestimation in the caries experience of the population. Another limitation of this methodology was that a patient-doctor rapport was not created due to non-interaction between the doctor and the patient. As enforced in the pedodontic treatment triangle for effective dental treatment for children, it is mandatory to have healthy interaction with both the parent and the child. This needs to be further incorporated into the teledentistry programs.

## Conclusions

School dental health programs have good accessibility and cause minimal disruption of schedules for the children but require the time and presence of a dentist, making them not very cost effective. Hence, this study was initiated to evaluate if dental caries could be imaged and diagnosed with images. The scores of dmft for children across the three age groups were consistent when calculated using the three different ways; based on these findings, the images made using an intraoral camera is a feasible alternative to a visual oral examination for caries screening in children between the age group of three to six years at school. This provides access to evaluate, assess, and monitor one of the most common afflictions of the oral cavity in children without the physical presence of the dentist. This technique promises to be a potent armamentarium in improving the access to dental care for children worldwide.
